# Synthesis, crystal structure and Hirshfeld surface analysis of (2-amino­benzo­thia­zole-κ*N*^3^)aqua­bis­(4-oxopent-2-en-2-olato-κ^2^*O*,*O*′)cobalt(II)

**DOI:** 10.1107/S2056989025008011

**Published:** 2025-09-11

**Authors:** Iroda Tojiboyeva, Sardor Murodov, Lola Makhmudova, Daminbek Ziyatov, Jamshid Ashurov, Shakhlo Daminova

**Affiliations:** a Tashkent State Medical University, Farobiy Street, 2, Almazar district, Tashkent, 100109, Uzbekistan; b National University of Uzbekistan named after Mirzo Ulugbek University Street 4 Tashkent 100174 Uzbekistan; c Uzbekistan-Japan Innovation Centre of Youth, University Street 2B, Tashkent 100095, Uzbekistan; d Institute of Bioorganic Chemistry, Academy of Sciences of Uzbekistan, Mirzo Ulugbek Street 83, 8 Tashkent 100125, Uzbekistan; Universidad de la Repüblica, Uruguay

**Keywords:** X-ray diffraction, Hirshfeld surface, heteroligand complex, h-bond chain, π–π stacking, crystal structure

## Abstract

The crystal structure of the title complex, [Co(C_5_H_7_O_2_)_2_(C_7_H_6_N_2_S)(H_2_O)], was determined in the triclinic space group *P*1. The central Co^II^ ion adopts a slightly distorted octa­hedral geometry. The unit cell consists of two complex mol­ecules connected *via* N—H⋯O and O—H⋯O hydrogen bonds along the [011] direction. Hirshfeld surface analysis revealed that the largest contributions to the crystal packing originate from H⋯H (51.8%), H⋯C/C⋯H (16.6%), O⋯H/H⋯O (12.4%), and H⋯S/S⋯H (8.8%) contacts.

## Abstract 2

The crystal structure of the cobalt complex [Co(C_5_H_7_O_2_)_2_(C_7_H_6_N_2_S)(H_2_O)] was determined in the triclinic space group *P*ī. The unit cell consists of two independent complex mol­ecules linked by N—H⋯O and O—H⋯O hydrogen bonds along the [011] direction. Hirshfeld surface analysis revealed that the largest contributions to the crystal packing originate from H⋯H, H⋯C/C⋯H, O⋯H/H⋯O, and H⋯S/S⋯H contacts.

## 1. Chemical context

In recent years, complex compounds based on ligands such as β-diketones and 2-amino­benzo­thia­zole have gained significant attention. β-Diketones, well known for their keto–enolic tautomerism (Tighadouini *et al.*, 2022 ▸), are present in a wide range of bioactive mol­ecules, serving both as structural scaffolds for complexation and as valuable agents with anti­oxidant properties. They have been investigated as potential therapeutic agents for treating hypertension, obesity, diabetes, neurological disorders, inflammatory and skin conditions, fibrosis, and arthritis (de Gonzalo & Alcantara 2021 ▸). Acetyl­acetonate (acac), a representative member of the β-diketone class, has been extensively studied as a ligand in metal–organic complexes (Pettinari *et al.*, 2003 ▸) and is well-established in preparative chemistry (Pradhan & Goyal 2015 ▸). In addition to its bioactive properties, it has been employed in fluorescence applications, for example in Eu(acac)_3_ (Kuz’mina & Eliseeva 2006 ▸). The use of β-diketones as ligands has also become pivotal in the chemistry of rare-earth metals (Duan *et al.*, 2022 ▸) and in the bidentate separation of certain radioactive isotopes of *d*-block metals, such as Cu, Co, and Ni (Caminati & Grabow 2006 ▸). Moreover, their ability to form stable chelates with actinide elements has made β-diketones highly relevant in the design of extraction agents for the reprocessing of spent nuclear fuel and the separation of uranium and other actinide species (Jabborova *et al.*, 2024 ▸).

2-Amino­benzo­thia­zole is a benzo­thia­zole derivative that serves as parent scaffold for numerous pharmaceuticals. Its enamine tautomerism influences its reactivity (Javahershenas *et al.*, 2024 ▸; Abdullayeva *et al.*, 2025 ▸). The widespread inter­est in amino­benzo­thia­zole cores has led to the development of a variety of synthetic methods, including the use of ammonium thio­cyanate, thio­urea, and condensation of *o*-haloanilines (Dadmal *et al.*, 2018 ▸). 6-Substitution of 2-amino­benzo­thia­zoles has been shown to yield compounds with notable *in vitro* anti­fungal activity (MIC 4–8 µg mL^−1^) against Candida species, exhibiting low toxicity towards THP-1 cells (Catalano *et al.*, 2013 ▸). Moreover, optically active 2-amino­benzo­thia­zole derivatives have demonstrated cytotoxic activity against EAC, MCF-7, and HeLa cells (IC50 = 10–30 µ*M*), inducing dose-dependent DNA damage, with IVe, IVf, and IVh exhibiting the highest activity (Manjula *et al.*, 2009 ▸). In this context, we have synthesized the complex (**I**) for further studies of its anti­microbial and anti­viral properties. The structural characteristics, including the three-dimensional mol­ecular geometry, hydrogen-bonding patterns, and Hirshfeld surface analyses, are discussed.
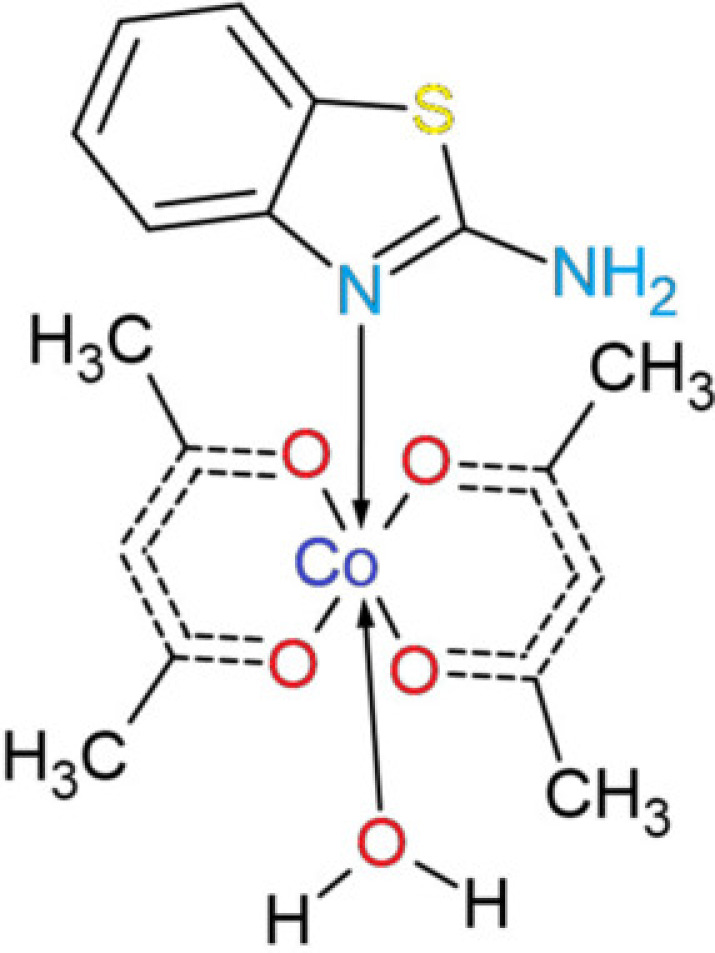


## 2. Structural commentary

**Figure 1:**
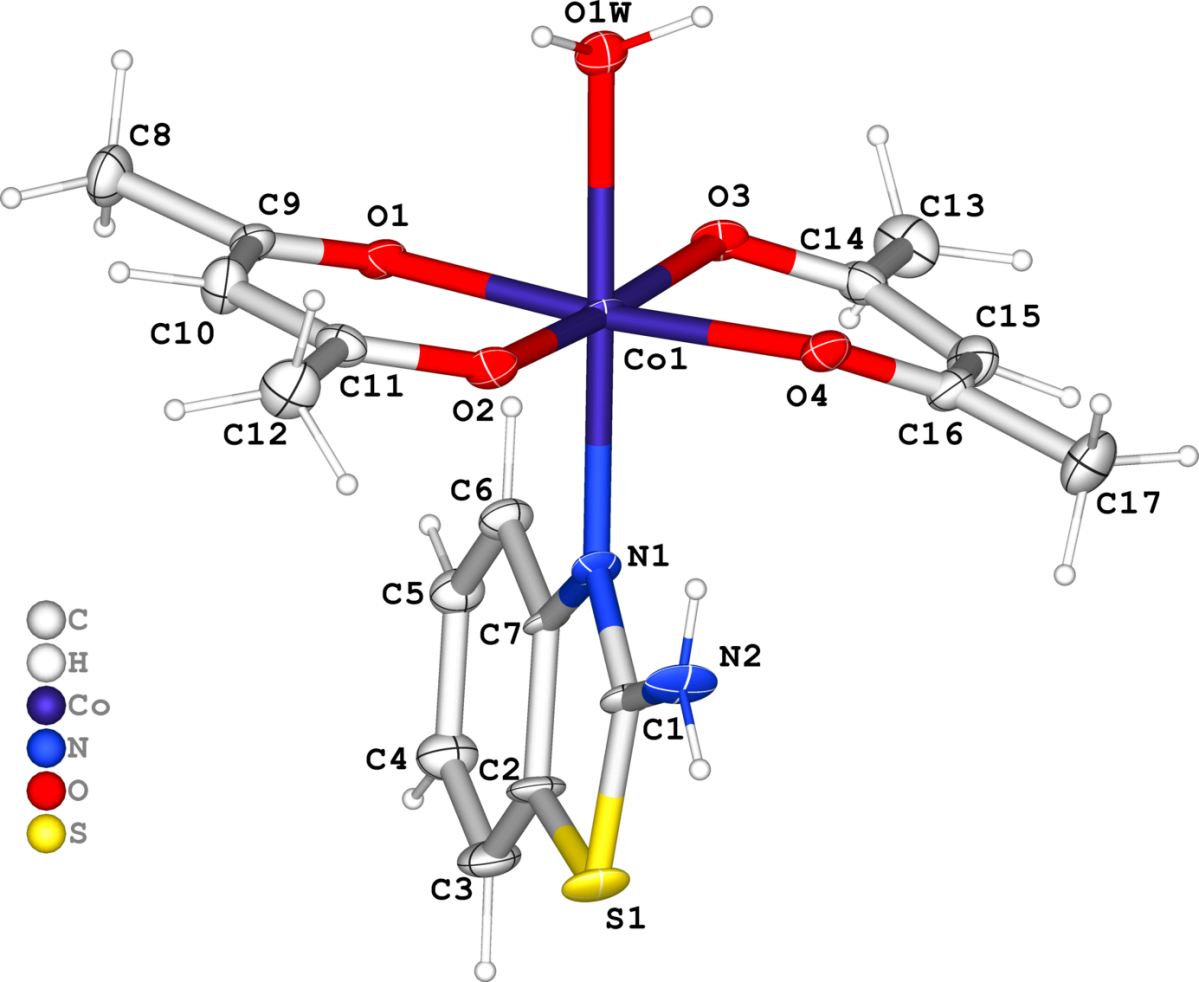
Asymmetric unit of the title compound with the atom-numbering scheme. Displacement ellipsoids for non-hydrogen atoms are drawn at the 50% probability level.

**Figure 2:**
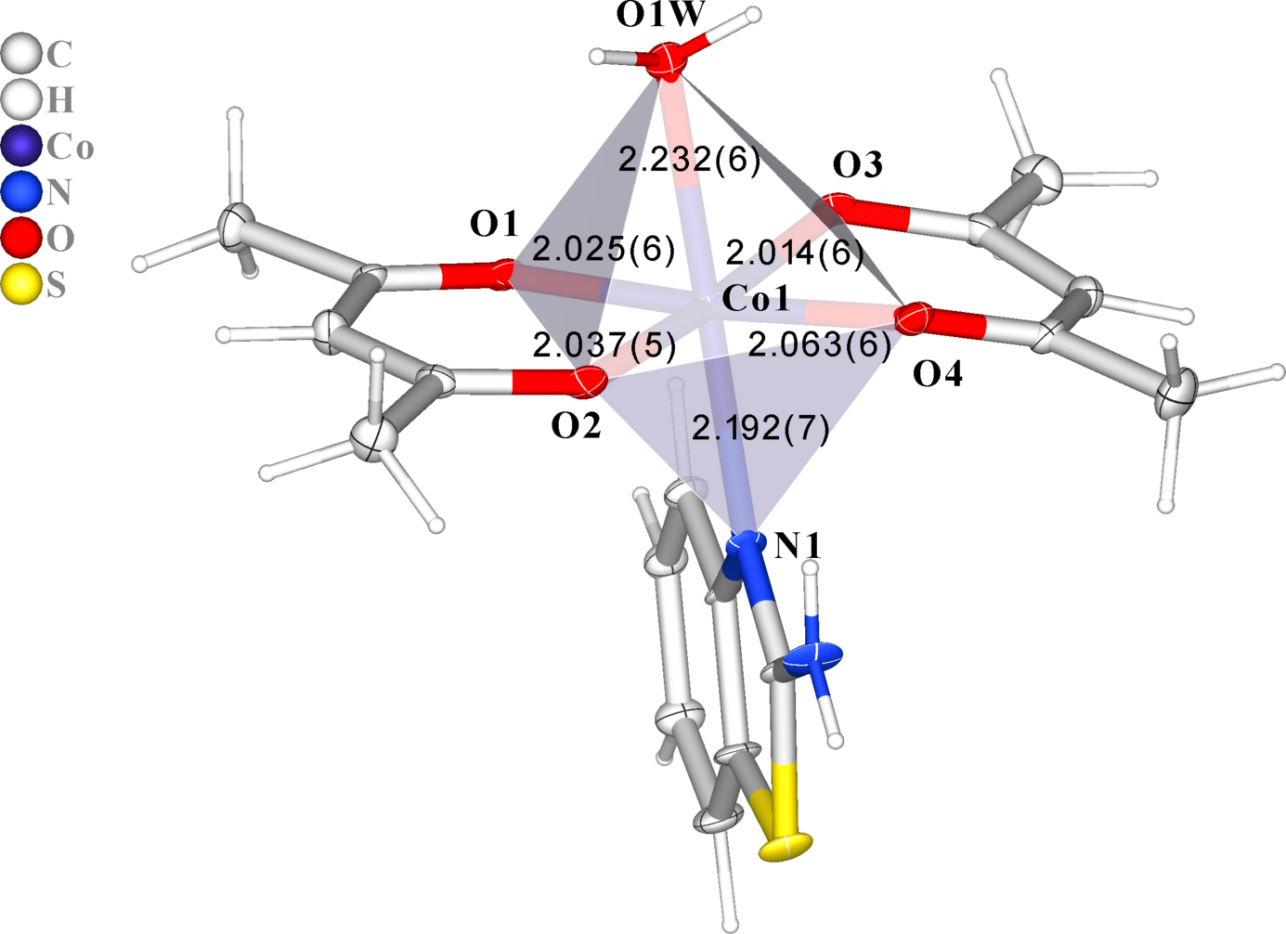
The octa­hedral coordination environment of the metal center in the title compound, with selected bond lengths indicated.

Complex (**I**) (Fig. 1 ▸) crystallizes in the triclinic space group *Pī*. The asymmetric unit of the heteroleptic complex comprises two acetyl­acetonate (acac) ligands, one 2–amino­benzo­thia­zole (ABT) mol­ecule, and one water mol­ecule coordinated to the Co^II^ center. The central Co^II^ ion adopts a slightly distorted octa­hedral geometry (Fig. 2 ▸) with a coordination number of six. The acetyl­acetonate ligands bind in a bidentate fashion through their carbonyl oxygen atoms. The axial positions are occupied by an *sp*^2^-hybridized nitro­gen atom from the ABT ligand and an oxygen atom of the water mol­ecule. The Co–ligand bond distances range from 2.014 (6) to 2.232 (6) Å (Table 1 ▸), indicating the presence of a Co^+2^ center, as Co^+3^ complexes generally display shorter bond distances (∼1.9–2.1 Å), especially for Co—O bonds. In the complex, the two acetyl­acetonate mol­ecules are nearly coplanar. The chelate angles O1—Co—O2 = 90.2 (2)° and O3—Co—O4 = 86.9 (2)° are typical for acetyl­acetonate ligands (Siddikova *et al.*, 2024 ▸, 2025 ▸), with a slight angular distortion (∼3.3°) suggesting some strain within the chelate rings (Co–O3–C14–C15–C16–O4). The least-squares plane defined by atoms O1, O2, O3, O4, O1*W*, N1 and Co has an r.m.s. deviation of 0.04 Å, with the Co atom displaced from this plane by 0.077 (2) Å.

The structural parameters of the obtained complex were further compared with the Co^II^ coordination compound reported by Thamilarasan *et al.* (2016 ▸), where a similar coordination environment comprises two acetyl­acetonate ligands, a water mol­ecule, and a pyridine ligand in the axial positions. In both structures, the cobalt atom exhibits a slightly distorted octa­hedral geometry. The Co—O(acac) bond lengths in the pyridine complex are 1.895–1.900 Å, which are somewhat shorter than those observed in complex (**I**) [2.014 (6)–2.064 (6) Å]. The longer distances can be attributed to the electronic nature of the ABT ligand and its steric effects. Similarly, the Co—N(ABT) bond length in the title structure is 2.192 (7) Å, which is significantly longer than Co—N(py) = 1.919 (2) Å, due to the greater steric and electronic saturation of the nitro­gen atom in ABT. The coordinated water mol­ecule also exhibits a longer Co—O bond in complex (**I**) [2.232 (6) Å *vs* 2.104 (2) Å], which may be associated with the overall octa­hedral distortion.

## 3. Supra­molecular features

**Figure 3:**
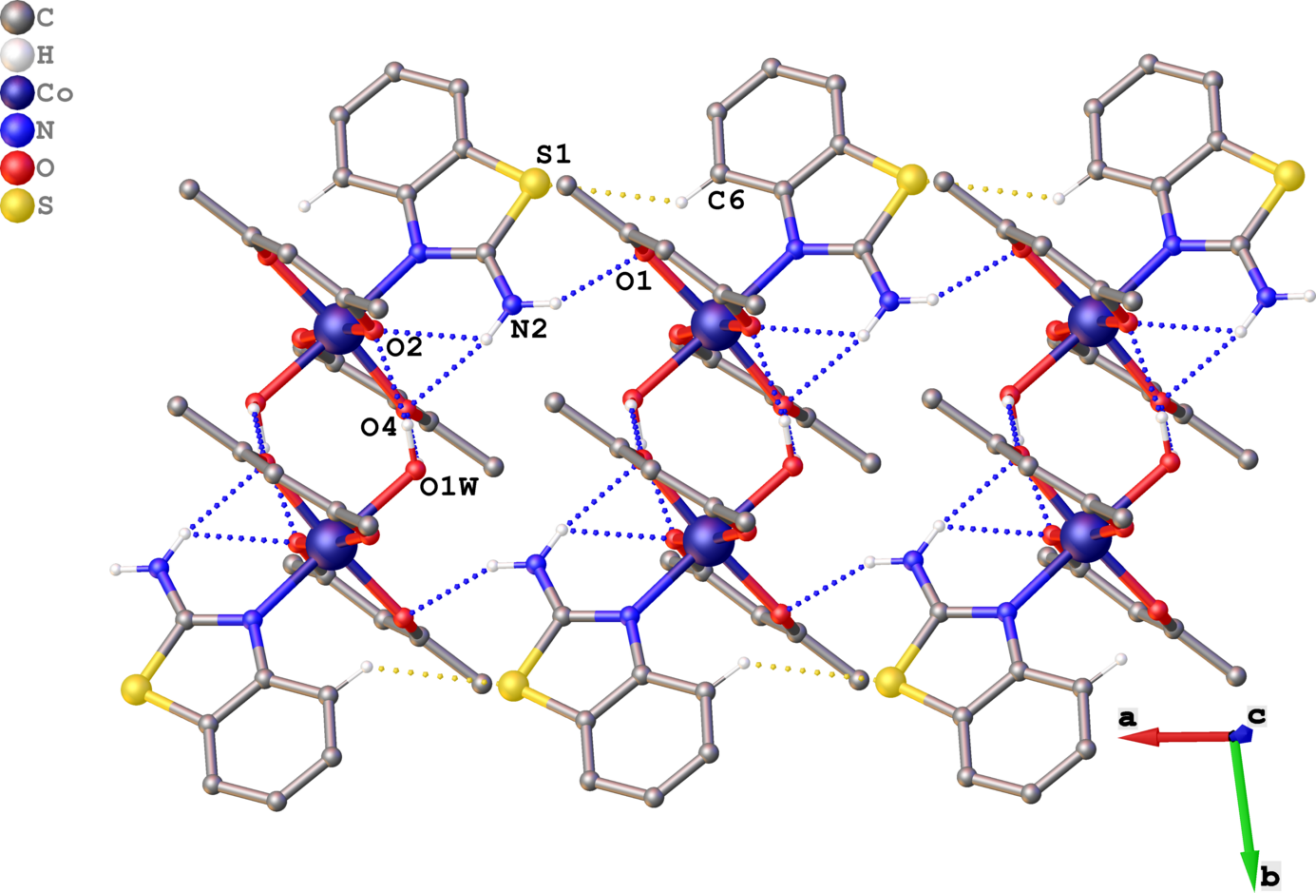
Supra­molecular structure of the title complex showing N—H⋯O and O—H⋯O hydrogen bonds (blue dashed lines) and non-classical C—H⋯S inter­actions (yellow dashed lines), forming chains along [01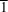]. Only hydrogen atoms involved in these inter­actions are shown.

In the crystal, several inter­molecular inter­actions are observed, including classical hydrogen bonds of the N—H⋯O and O—H⋯O types, as well as a weak C—H⋯S inter­action (Table 2 ▸). In particular, O1*W*—H1*Wa*⋯O2, O1*W*—H1*Wb*⋯O4, N2—H2*b*⋯O1, and C6—H6⋯S1 can be distinguished, which are organized into chains oriented along the [01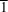] direction (Fig. 3 ▸). Notably, the coordinated water mol­ecule plays an essential role as a ligand, participating in the formation of two inter­molecular hydrogen bonds, O1*W*—H1*Wa*⋯O2 [2.808 (8) Å] and O1*W*—H1*Wb*⋯O4 [2.820 (8) Å].

The crystal packing is also consolidated by intra­molecular hydrogen bonds (Table 2 ▸). Specifically, two N—H⋯O inter­actions, N2—H2*a*⋯O2 [3.060 (10) Å] and N2—H2*a*⋯O4 [3.028 (10) Å], as well as one C—H⋯O inter­action, C6—H6⋯O3 [3.189 (11) Å], are observed.

The structure of the complex further exhibits pronounced *π–π* stacking inter­actions between the aromatic rings of the benzo­thia­zole fragments of adjacent mol­ecules. These inter­actions are oriented along the [01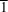] direction and contribute to the densification of the packing.

**Figure 4:**
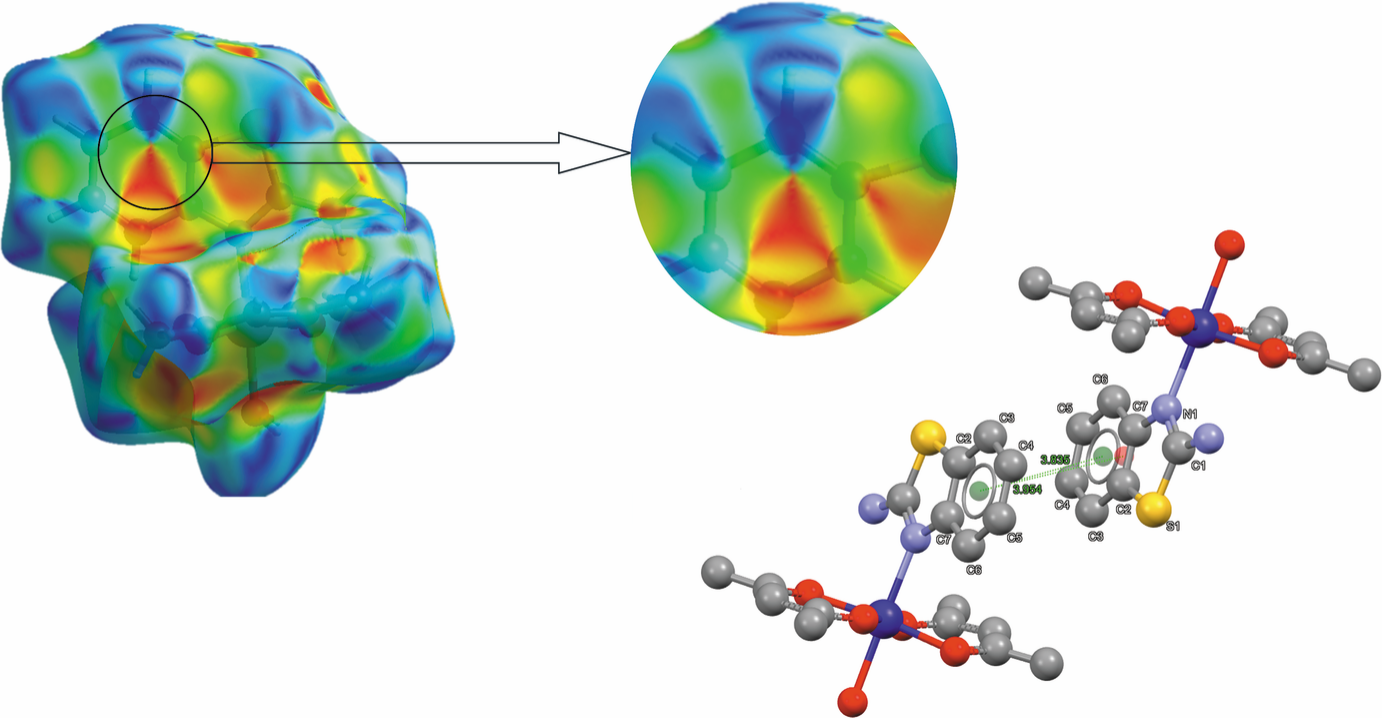
π–π stacking inter­actions in the crystal structure of the title complex. The Hirshfeld surface mapped with the shape-index clearly shows adjacent red and blue triangular patches (top), which indicate the presence of π–π contacts. These inter­actions occur between the 2-amino­benzo­thia­zole rings of neighboring mol­ecules, and the corresponding inter­centroid distances are given (bottom).

The aromatic system of the benzo­thia­zole ligand consists of a fused heterocyclic ring system (*Cg*5: N1/S1/C1–C7), incorporating both a benzene ring (*Cg*4: C2–C7) and a thia­zole ring. Two types of inter­actions are observed (Fig. 4 ▸): *Cg*4⋯*Cg*4, with a centroid–centroid distance of 3.835 (5) Å between benzene rings, and *Cg*4⋯*Cg*5, with a centroid centroid–centroid of 3.954 (5) Å between the benzene and the entire benzo­thia­zole ring system. The dihedral angles between the respective ring planes are small [< 10°; *Cg*4⋯*Cg*4 = 0.0 (4)°, *Cg*4⋯*Cg*5 = 0.2 (4)°], indicating a parallel, face-to-face (π–π) stacking of the π-systems and a favorable orbital overlap geometry. These contacts, together with the hydrogen-bonding network, contribute to the formation of layered motifs in the crystal packing.

## 4. Hirshfeld Surface

**Figure 5:**
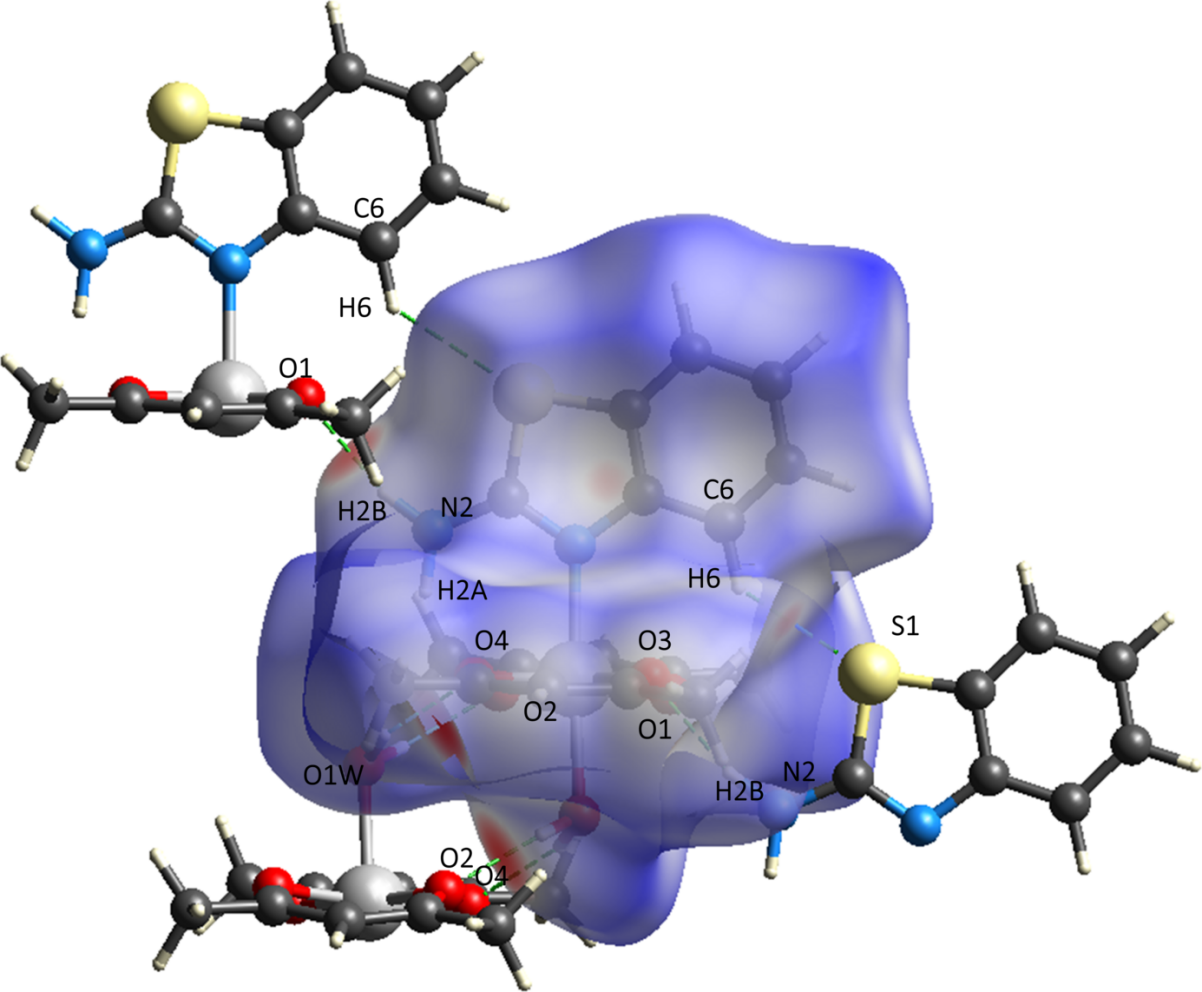
Hirshfeld surface of the title complex mapped over *d*_norm_, highlighting close inter­molecular contacts as red spots corresponding to regions of strong hydrogen-bonding inter­actions.

**Figure 6:**
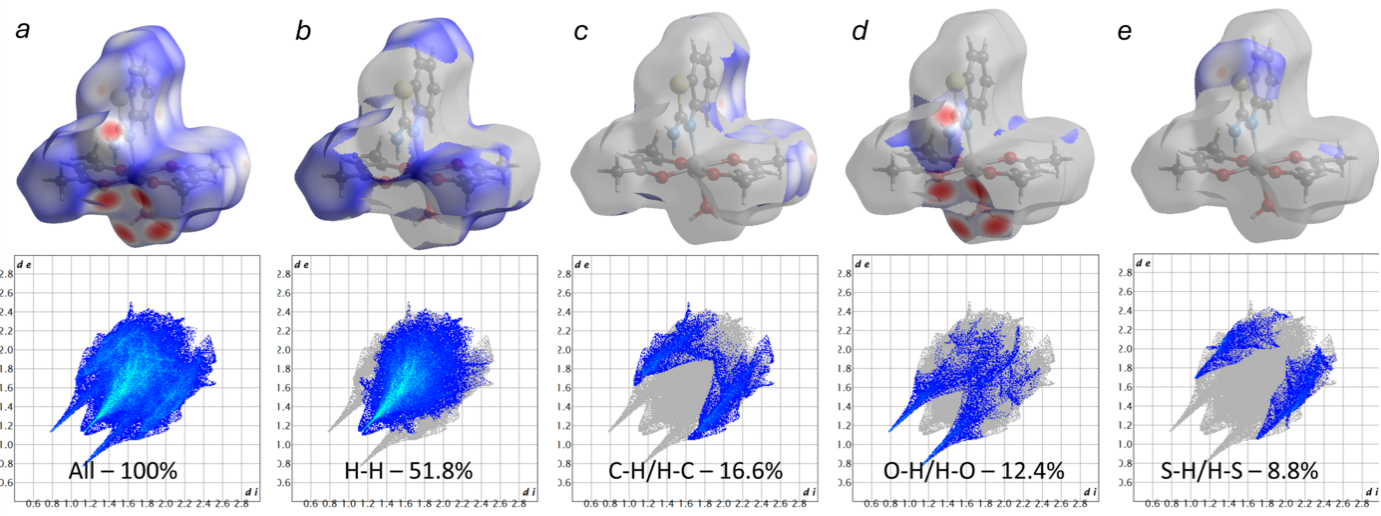
Full two-dimensional fingerprint plots of the title compound, mapped over *d*_norm_, showing all inter­actions (*a*) and delineated into selected inter­actions: (*b*) H⋯H, (*c*) C⋯H/H⋯C, (*d*) O⋯H/H⋯O, and (*e*) S⋯H/H⋯S, together with their relative contributions to the Hirshfeld surface.

The Hirshfeld surface (HS) and the corresponding two-dimensional fingerprint plots were calculated using *CrystalExplorer 21.5* (Spackman *et al.*, 2021 ▸). In the *d*_norm_ map (Fig. 5 ▸), intense red regions indicate inter­molecular contacts shorter than the sum of the van der Waals radii, whereas blue regions correspond to longer contacts. White areas represent contacts close to the sum of these radii (Venkatesan *et al.*, 2016 ▸). The overall fingerprint plot (Fig. 6 ▸*a*) shows that the largest contribution to the surface inter­actions arises from H⋯H contacts, accounting for 51.8% (Fig. 6 ▸*b*). This is typical for organic mol­ecules with a high degree of hydrogen saturation and indicates dense mol­ecular packing. The O⋯H/H⋯O contacts (12.4%, Fig. 6 ▸*d*) reflect the presence of both classical (O—H⋯O) and non-classical (N—H⋯O) hydrogen bonds, consistent with the crystal packing data (Table 2 ▸, Fig. 3 ▸). On the *d*_norm_ surface, these inter­actions appear as intense red spots, highlighting their significant role in consolidating the structure. The H⋯C/C⋯H (16.6%, Fig. 6 ▸*c*) and H⋯S/S⋯H (8.8%, Fig. 6 ▸*e*) contacts correspond to weak van der Waals inter­actions and C—H⋯S contacts, previously identified as potential non-classical hydrogen bonds. Although the qu­anti­tative contribution of *π–π* inter­actions (through C⋯C contacts) is relatively small (1.9%), the shape-index surface (Fig. 4 ▸) clearly displays alternating red and blue patches on the aromatic regions, characteristic of *π–π* stacking. This agrees with the structural data (Fig. 4 ▸), where centroid–centroid distances of 3.835 (5) and 3.954 (5) Å are observed between the benzo­thia­zole fragments.

A comparison with the related complex [Co(acac)_2_(py)(H_2_O)] (Thamilarasan *et al.*, 2016 ▸) shows that *π⋯π* contacts are more pronounced in complex I, whereas in the pyridine analogue the packing is primarily consolidated by O–H⋯O hydrogen bonds. This emphasizes the importance of stacking inter­actions in consolidating the present structure. In the benzo­thia­zole complex reported by Srhir *et al.* (2020 ▸), the contributions of H⋯H, O⋯H, H⋯C, and H⋯S contacts were 47.0%, 16.9%, 8.0%, and 7.6%, respectively, with *π–π* stacking visually noted but not qu­anti­tatively discussed. In the Cu^I^-benzimidazole complex, H⋯H contacts accounted for 34.6%, while C⋯C (*π–π*) inter­actions were minimal (Chooto *et al.*, 2022 ▸). In contrast, the significant contribution of *π–π* stacking in our case, confirmed both visually (shape index) and structurally (centroid–centroid distances of 3.835 (5) Å and 3.954 (5) Å), differs from the less pronounced cases reported in the literature. This highlights the uniqueness of the packing in complex I, where not only hydrogen bonds but also aromatic stacking inter­actions play a substantial role.

Thus, the Hirshfeld surface analysis not only confirms the inter­molecular contacts observed in the structural model but also enhances the understanding of the crystal packing, demonstrating the contributions of both strong (hydrogen bonds) and weak (*π–π*, C—H⋯S) inter­actions.

## 5. Database survey

A survey of the Cambridge Structural Database (CSD2024.2.0; Groom *et al.*, 2016 ▸) revealed three closely related structures containing the ABT moiety. Approximately 60 ABT-containing structures were identified, including octa­hedral complexes where ABT and acetyl­acetonate ligands coordinate as bidentate ligands *via* oxygen atoms, with ABT binding through its nitro­gen site [CSD refcodes: SUSWIN (Hai-Bin Gu *et al.*, 2010 ▸) and SUVTEI (Sieroń & Bukowska-Strzyżewska 1999 ▸)]. Other examples of ABT–ligand complexes can be found in refcodes ABODIG (Gao *et al.*, 2011 ▸), CAZJIY (Gu *et al.*, 2012 ▸), and GARSEZ (Kim *et al.*, 2012 ▸). The acetyl­acetonate motif appears in roughly 20 structures, both as the sole bidentate ligand [refcode: ACACCE (Matković & Grdenić, 1963 ▸)] and in heteroleptic environments [refcode: ACNIET10 (Pfluger *et al.*, 1973 ▸)].

## 6. Synthesis and crystallization

The following solutions were prepared: (*a*) ethanol solution of CoCl_2_·6H_2_O (0.238 g, ∼1.0 mmol), (*b*) ethanol solution of 2-amino­benzo­thia­zole (0.300 g, ∼2.0 mmol) and (*c*) acetyl­acetonate (0.2 mmol; *V* = 0.0205 mL, ρ = 0.975 g mL^−1^). Solution (*a*) was added to solution (*b*) and stirred for 30 minutes at room temperature on a magnetic stirrer. After this, solution (*c*) was added dropwise and stirred for 12 h, yielding a blue crystalline precipitate. The precipitate was filtered, washed several times with ethanol, and dried in air. Since the resulting material is readily soluble in DMF, it was recrystallized from this solvent to obtain well-formed, blue single crystals suitable for structural and further physicochemical studies.
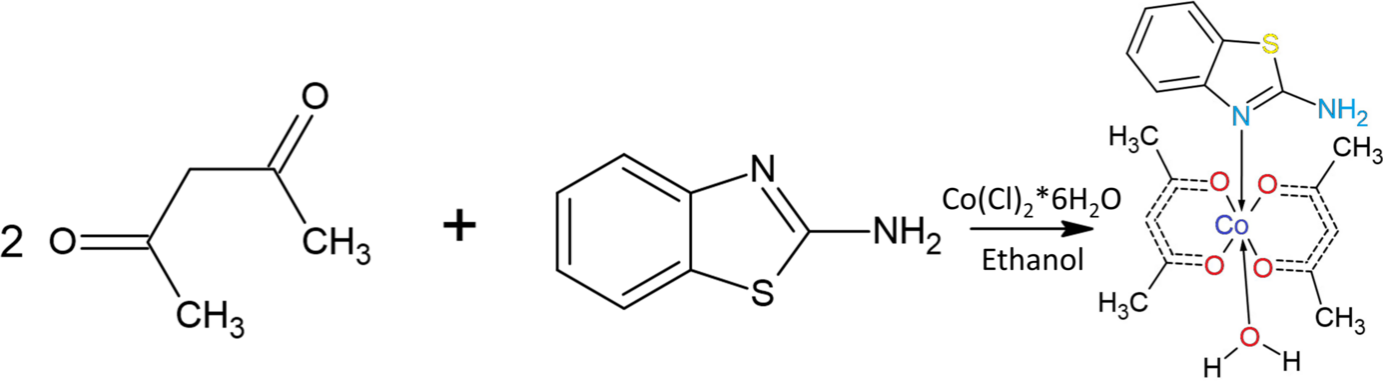


## 7. Refinement details

Crystal data, data collection and structure refinement details are summarized in Table 3 ▸. C–bound hydrogen atoms were placed geometrically and treated as riding atoms, with C—H = 0.93 Å (aromatic), 0.96 Å (meth­yl), and 0.97 Å (methyl­ene). *U*_iso_(H) was set to 1.5*U*_eq_(C) for methyl hydrogen atoms and 1.2*U*_eq_(C) otherwise. The hy­droxy hydrogen was located at O—H = 0.84 Å and water hydrogen atoms were positioned with O—H = 0.82 Å and refined with *U*_iso_(H) = 1.5*U_eq_*(O).

## Supplementary Material

Crystal structure: contains datablock(s) I. DOI: 10.1107/S2056989025008011/ny2015sup1.cif

CCDC reference: 2486714

Additional supporting information:  crystallographic information; 3D view; checkCIF report

Additional supporting information:  crystallographic information; 3D view; checkCIF report

## Figures and Tables

**Table 1:** Selected geometric parameters (Å, °)

Co1—O1	2.025 (6)	Co1—O4	2.063 (6)
Co1—O2	2.037 (5)	Co1—O1*W*	2.232 (6)
Co1—O3	2.014 (6)	Co1—N1	2.192 (7)
O2—Co1—O1	90.2 (2)	O4—Co1—O3	86.9 (2)

Symmetry code: (i) 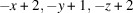.

**Table 2:** Hydrogen-bond geometry (Å, °)

*D*—H⋯*A*	*D*—H	H⋯*A*	*D*⋯*A*	*D*—H⋯*A*
O1*W*—H1*Wa*⋯O2^ii^	0.92 (2)	1.98 (3)	2.808 (8)	148 (5)
O1*W*—H1*Wb*⋯O4^ii^	0.92 (1)	1.97 (3)	2.820 (8)	154 (5)
N2—H2*b*⋯O1^iii^	0.86 (1)	2.29 (1)	3.074 (10)	151 (1)
C6—H6⋯S1^iv^	0.93 (1)	2.85 (1)	3.568 (10)	135 (1)
N2—H2*a*⋯O2	0.86 (1)	2.48 (1)	3.060 (10)	126 (1)
N2—H2*a*⋯O4	0.86 (1)	2.38 (1)	3.028 (10)	132 (1)
C6—H6⋯O3	0.93 (1)	2.53 (1)	3.189 (11)	128 (1)

Symmetry codes: (ii) 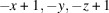; (iii) 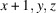; (iv) 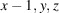.

**Table 3:** Experimental details

Crystal data	
Chemical formula	[Co(C_5_H_7_O_2_)_2_(C_7_H_6_N_2_S)(H_2_O)]
*M*_r_	425.37
Crystal system, space group	Triclinic, *P* 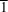
Temperature (K)	293
*a*, *b*, *c* (Å)	7.3803 (4), 11.1947 (8), 12.4796 (8)
α, β, γ (°)	95.803 (5), 105.633 (5), 95.875 (5)
*V* (Å^3^)	978.76 (11)
*Z*	2
Radiation type	Cu *K*α
μ (mm^−1^)	8.13
Crystal size (mm)	0.41 × 0.24 × 0.15

Data collection	
Diffractometer	XtaLAB Synergy, Single source at home/near, HyPix3000
Absorption correction	Multi-scan (*CrysAlis PRO*; Rigaku OD, 2025 ▸)
*T*_min_, *T*_max_	0.271, 1.000
No. of measured, independent and observed [*I* ≥ 2u(*I*)] reflections	8259, 3511, 2051
*R*_int_	0.125
(sin θ/λ)_max_ (Å^−1^)	0.601

Refinement	
*R*[*F*^2^ > 2σ(*F*^2^)], *wR*(*F*^2^), *S*	0.116, 0.315, 0.98
No. of reflections	3511
No. of parameters	241
H-atom treatment	H atoms treated by a mixture of independent and constrained refinement
Δρ_max_, Δρ_min_ (e Å^−3^)	1.62, −1.94

Computer programs: *CrysAlis PRO* (Rigaku OD, 2025 ▸), *SHELXT2018/2* (Sheldrick, 2015 ▸), *OLEX2.refine*(Bourhis *et al.*, 2015 ▸)and *OLEX2* (Dolomanov *et al.*, 2009 ▸).

## supplementary crystallographic information

### (2-Aminobenzothiazole-κN3)aquabis(4-oxopent-2-en-2-olato-κ2O,O')cobalt(II) Crystal data

**Table:**

[Co(C_5_H_7_O_2_)_2_(C_7_H_6_N_2_S)(H_2_O)]	*Z* = 2
*M**_r_* = 425.37	*F*(000) = 439.691
Triclinic, *P*1	*D*_x_ = 1.443 Mg m^−^^3^
*a* = 7.3803 (4) Å	Cu *K*α radiation, λ = 1.54184 Å
*b* = 11.1947 (8) Å	Cell parameters from 2947 reflections
*c* = 12.4796 (8) Å	θ = 3.6–67.9°
α = 95.803 (5)°	µ = 8.13 mm^−^^1^
β = 105.633 (5)°	*T* = 293 K
γ = 95.875 (5)°	Plate, clear greenish blue
*V* = 978.76 (11) Å^3^	0.41 × 0.24 × 0.15 mm

### (2-Aminobenzothiazole-κN3)aquabis(4-oxopent-2-en-2-olato-κ2O,O')cobalt(II) Data collection

**Table:**

XtaLAB Synergy, Single source at home/near, HyPix3000 diffractometer	3511 independent reflections
Radiation source: micro-focus sealed X-ray tube, PhotonJet (Cu) X-ray Source	2051 reflections with *I*≥ 2u(*I*)
Mirror monochromator	*R*_int_ = 0.125
Detector resolution: 10.0000 pixels mm^-1^	θ_max_ = 68.0°, θ_min_ = 3.7°
ω scans	*h* = −8→8
Absorption correction: multi-scan (CrysAlisPro; Rigaku OD, 2025)	*k* = −13→13
*T*_min_ = 0.271, *T*_max_ = 1.000	*l* = −14→14
8259 measured reflections	

### (2-Aminobenzothiazole-κN3)aquabis(4-oxopent-2-en-2-olato-κ2O,O')cobalt(II) Refinement

**Table:**

Refinement on *F*^2^	0 restraints
Least-squares matrix: full	34 constraints
*R*[*F*^2^ > 2σ(*F*^2^)] = 0.116	H atoms treated by a mixture of independent and constrained refinement
*wR*(*F*^2^) = 0.315	*w* = 1/[σ^2^(*F*_o_^2^) + (0.2*P*)^2^] where *P* = (*F*_o_^2^ + 2*F*_c_^2^)/3
*S* = 0.98	(Δ/σ)_max_ = 0.001
3511 reflections	Δρ_max_ = 1.62 e Å^−^^3^
241 parameters	Δρ_min_ = −1.94 e Å^−^^3^

### (2-Aminobenzothiazole-κN3)aquabis(4-oxopent-2-en-2-olato-κ2O,O')cobalt(II) Fractional atomic coordinates and isotropic or equivalent isotropic displacement parameters (Å^2^)

**Table:**

	*x*	*y*	*z*	*U*_iso_*/*U*_eq_	
Co1	0.56729 (15)	0.18957 (11)	0.63199 (10)	0.0382 (5)	
S1	1.1564 (3)	0.4410 (2)	0.7783 (2)	0.0630 (8)	
O1	0.3971 (7)	0.3172 (5)	0.5851 (5)	0.0446 (14)	
O2	0.6355 (7)	0.1835 (5)	0.4840 (5)	0.0455 (14)	
O3	0.4835 (8)	0.1762 (6)	0.7716 (5)	0.0488 (15)	
O4	0.7204 (7)	0.0487 (5)	0.6747 (5)	0.0464 (14)	
O1W	0.3062 (8)	0.0603 (5)	0.5438 (5)	0.0471 (14)	
H1Wa	0.314 (4)	−0.0177 (15)	0.560 (5)	0.071 (2)*	
H1Wb	0.288 (6)	0.048 (5)	0.4675 (7)	0.071 (2)*	
N1	0.8166 (8)	0.3210 (6)	0.7218 (6)	0.0421 (16)	
N2	1.0354 (10)	0.2282 (7)	0.6473 (7)	0.064 (2)	
H2a	0.9500 (10)	0.1682 (7)	0.6135 (7)	0.077 (3)*	
H2b	1.1488 (10)	0.2297 (7)	0.6410 (7)	0.077 (3)*	
C1	0.9920 (10)	0.3194 (8)	0.7096 (7)	0.044 (2)	
C2	0.9855 (10)	0.5035 (8)	0.8315 (8)	0.051 (2)	
C3	1.0050 (13)	0.6090 (10)	0.9016 (9)	0.067 (3)	
H3	1.1209 (13)	0.6590 (10)	0.9276 (9)	0.080 (3)*	
C4	0.8462 (14)	0.6398 (9)	0.9332 (9)	0.062 (3)	
H4	0.8538 (14)	0.7115 (9)	0.9798 (9)	0.074 (3)*	
C5	0.6797 (13)	0.5631 (8)	0.8948 (7)	0.051 (2)	
H5	0.5754 (13)	0.5845 (8)	0.9169 (7)	0.062 (3)*	
C6	0.6573 (12)	0.4556 (8)	0.8249 (8)	0.049 (2)	
H6	0.5414 (12)	0.4056 (8)	0.8007 (8)	0.059 (3)*	
C7	0.8148 (9)	0.4243 (7)	0.7918 (6)	0.0361 (17)	
C8	0.1966 (15)	0.4309 (10)	0.4689 (10)	0.068 (3)	
H8a	0.0747 (19)	0.3849 (15)	0.459 (6)	0.103 (4)*	
H8b	0.222 (6)	0.493 (4)	0.532 (3)	0.103 (4)*	
H8c	0.197 (8)	0.467 (6)	0.403 (4)	0.103 (4)*	
C9	0.3480 (11)	0.3479 (8)	0.4899 (7)	0.0416 (19)	
C10	0.4237 (13)	0.3163 (9)	0.4001 (8)	0.055 (2)	
H10	0.3783 (13)	0.3500 (9)	0.3346 (8)	0.066 (3)*	
C11	0.5616 (12)	0.2383 (8)	0.4010 (7)	0.045 (2)	
C12	0.6302 (15)	0.2173 (11)	0.2997 (8)	0.067 (3)	
H12a	0.764 (3)	0.212 (7)	0.3225 (8)	0.100 (4)*	
H12b	0.563 (8)	0.143 (4)	0.255 (3)	0.100 (4)*	
H12c	0.608 (10)	0.284 (4)	0.257 (3)	0.100 (4)*	
C13	0.5093 (18)	0.1638 (11)	0.9600 (9)	0.075 (3)	
H13a	0.546 (11)	0.2483 (19)	0.989 (4)	0.112 (5)*	
H13b	0.374 (2)	0.145 (7)	0.9404 (17)	0.112 (5)*	
H13c	0.566 (9)	0.116 (6)	1.016 (3)	0.112 (5)*	
C14	0.5743 (13)	0.1363 (8)	0.8595 (7)	0.049 (2)	
C15	0.7274 (14)	0.0702 (9)	0.8642 (7)	0.056 (2)	
H15	0.7923 (14)	0.0520 (9)	0.9342 (7)	0.067 (3)*	
C16	0.7904 (12)	0.0298 (8)	0.7736 (7)	0.048 (2)	
C17	0.9479 (16)	−0.0499 (12)	0.7911 (10)	0.082 (4)	
H17a	0.915 (6)	−0.117 (4)	0.732 (4)	0.122 (5)*	
H17b	1.064 (3)	−0.003 (2)	0.791 (7)	0.122 (5)*	
H17c	0.964 (9)	−0.080 (7)	0.862 (4)	0.122 (5)*	

### (2-Aminobenzothiazole-κN3)aquabis(4-oxopent-2-en-2-olato-κ2O,O')cobalt(II) Atomic displacement parameters (Å^2^)

**Table:**

	*U*^11^	*U*^22^	*U*^33^	*U*^12^	*U*^13^	*U*^23^
Co1	0.0157 (7)	0.0496 (9)	0.0512 (8)	0.0102 (5)	0.0120 (5)	0.0035 (6)
S1	0.0164 (10)	0.0830 (19)	0.0864 (17)	−0.0014 (10)	0.0164 (10)	−0.0001 (14)
O1	0.025 (3)	0.052 (4)	0.059 (4)	0.008 (2)	0.015 (2)	0.008 (3)
O2	0.028 (3)	0.061 (4)	0.054 (3)	0.008 (3)	0.022 (3)	0.005 (3)
O3	0.027 (3)	0.064 (4)	0.061 (4)	0.006 (3)	0.024 (3)	0.003 (3)
O4	0.030 (3)	0.055 (4)	0.053 (3)	0.005 (3)	0.010 (3)	0.007 (3)
O1W	0.037 (3)	0.050 (4)	0.055 (3)	0.010 (3)	0.016 (3)	0.000 (3)
N1	0.016 (3)	0.048 (4)	0.070 (4)	0.012 (3)	0.021 (3)	0.010 (3)
N2	0.022 (4)	0.073 (6)	0.103 (6)	0.006 (3)	0.037 (4)	−0.011 (5)
C1	0.015 (4)	0.061 (6)	0.056 (5)	0.000 (3)	0.012 (3)	0.003 (4)
C2	0.016 (4)	0.055 (6)	0.075 (6)	−0.007 (3)	0.008 (4)	−0.001 (5)
C3	0.034 (5)	0.064 (7)	0.084 (7)	−0.012 (5)	−0.003 (5)	−0.002 (6)
C4	0.049 (6)	0.060 (7)	0.074 (7)	0.004 (5)	0.018 (5)	−0.003 (5)
C5	0.042 (5)	0.053 (6)	0.057 (5)	0.007 (4)	0.014 (4)	−0.001 (4)
C6	0.028 (4)	0.051 (5)	0.069 (6)	0.009 (4)	0.015 (4)	0.002 (4)
C7	0.009 (3)	0.050 (5)	0.046 (4)	0.003 (3)	0.003 (3)	0.004 (4)
C8	0.060 (7)	0.070 (7)	0.083 (7)	0.033 (6)	0.020 (6)	0.029 (6)
C9	0.025 (4)	0.047 (5)	0.054 (5)	0.006 (3)	0.012 (3)	0.011 (4)
C10	0.056 (6)	0.066 (6)	0.054 (5)	0.023 (5)	0.026 (5)	0.020 (5)
C11	0.044 (5)	0.050 (5)	0.043 (5)	−0.003 (4)	0.020 (4)	0.004 (4)
C12	0.061 (7)	0.089 (8)	0.064 (6)	0.019 (6)	0.034 (5)	0.013 (5)
C13	0.090 (9)	0.078 (8)	0.055 (6)	0.003 (6)	0.025 (6)	−0.002 (5)
C14	0.047 (5)	0.056 (6)	0.045 (5)	−0.006 (4)	0.016 (4)	0.009 (4)
C15	0.053 (6)	0.070 (7)	0.041 (5)	0.005 (5)	0.010 (4)	0.012 (4)
C16	0.033 (4)	0.057 (6)	0.049 (5)	0.007 (4)	0.003 (4)	0.009 (4)
C17	0.059 (7)	0.095 (9)	0.089 (8)	0.034 (6)	0.007 (6)	0.023 (7)

### (2-Aminobenzothiazole-κN3)aquabis(4-oxopent-2-en-2-olato-κ2O,O')cobalt(II) Geometric parameters (Å, º)

**Table:**

Co1—O1	2.025 (6)	C5—C6	1.380 (12)
Co1—O2	2.037 (5)	C6—H6	0.9300
Co1—O3	2.014 (6)	C6—C7	1.399 (10)
Co1—O4	2.063 (6)	C8—H8a	0.9600
Co1—O1W	2.232 (6)	C8—H8b	0.9600
Co1—N1	2.192 (7)	C8—H8c	0.9600
S1—C1	1.710 (8)	C8—C9	1.511 (12)
S1—C2	1.749 (9)	C9—C10	1.410 (12)
O1—C9	1.241 (10)	C10—H10	0.9300
O2—C11	1.280 (10)	C10—C11	1.406 (12)
O3—C14	1.271 (10)	C11—C12	1.489 (11)
O4—C16	1.252 (9)	C12—H12a	0.9600
O1W—H1Wa	0.9212	C12—H12b	0.9600
O1W—H1Wb	0.9181	C12—H12c	0.9600
N1—C1	1.345 (9)	C13—H13a	0.9600
N1—C7	1.381 (10)	C13—H13b	0.9600
N2—H2a	0.8600	C13—H13c	0.9600
N2—H2b	0.8600	C13—C14	1.475 (13)
N2—C1	1.338 (11)	C14—C15	1.404 (13)
C2—C3	1.368 (13)	C15—H15	0.9300
C2—C7	1.401 (10)	C15—C16	1.386 (12)
C3—H3	0.9300	C16—C17	1.520 (13)
C3—C4	1.397 (14)	C17—H17a	0.9600
C4—H4	0.9300	C17—H17b	0.9600
C4—C5	1.363 (13)	C17—H17c	0.9600
C5—H5	0.9300		
			
Cg4···Cg4	0.0 (4)	Cg4···Cg4^i^	3.835 (5)
Cg5···Cg5	0.2 (4)	Cg4···Cg5^i^	3.954 (5)
			
O2—Co1—O1	90.2 (2)	C7—C6—H6	121.1 (5)
O3—Co1—O1	92.1 (2)	C2—C7—N1	116.5 (6)
O3—Co1—O2	173.4 (2)	C6—C7—N1	125.3 (7)
O4—Co1—O1	175.1 (2)	C6—C7—C2	118.3 (8)
O4—Co1—O2	90.3 (2)	H8b—C8—H8a	109.5
O4—Co1—O3	86.9 (2)	H8c—C8—H8a	109.5
O1W—Co1—O1	83.8 (2)	H8c—C8—H8b	109.5
O1W—Co1—O2	88.3 (2)	C9—C8—H8a	109.5
O1W—Co1—O3	85.8 (2)	C9—C8—H8b	109.5
O1W—Co1—O4	91.4 (2)	C9—C8—H8c	109.5
N1—Co1—O1	94.5 (2)	C8—C9—O1	116.8 (8)
N1—Co1—O2	93.3 (2)	C10—C9—O1	126.5 (8)
N1—Co1—O3	92.7 (2)	C10—C9—C8	116.6 (8)
N1—Co1—O4	90.3 (2)	H10—C10—C9	117.1 (5)
N1—Co1—O1W	177.7 (2)	C11—C10—C9	125.8 (8)
C2—S1—C1	90.0 (4)	C11—C10—H10	117.1 (5)
C9—O1—Co1	125.9 (5)	C10—C11—O2	124.6 (7)
C11—O2—Co1	126.1 (5)	C12—C11—O2	116.6 (8)
C14—O3—Co1	126.1 (5)	C12—C11—C10	118.8 (8)
C16—O4—Co1	123.9 (6)	H12a—C12—C11	109.5
H1Wa—O1W—Co1	113.5	H12b—C12—C11	109.5
H1Wb—O1W—Co1	111.9	H12b—C12—H12a	109.5
H1Wb—O1W—H1Wa	100.7	H12c—C12—C11	109.5
C1—N1—Co1	125.3 (6)	H12c—C12—H12a	109.5
C7—N1—Co1	125.3 (4)	H12c—C12—H12b	109.5
C7—N1—C1	109.1 (7)	H13b—C13—H13a	109.5
H2b—N2—H2a	120.0	H13c—C13—H13a	109.5
C1—N2—H2a	120.0	H13c—C13—H13b	109.5
C1—N2—H2b	120.0	C14—C13—H13a	109.5
N1—C1—S1	116.1 (6)	C14—C13—H13b	109.5
N2—C1—S1	121.9 (6)	C14—C13—H13c	109.5
N2—C1—N1	122.0 (7)	C13—C14—O3	115.6 (9)
C3—C2—S1	128.8 (6)	C15—C14—O3	123.9 (8)
C7—C2—S1	108.3 (6)	C15—C14—C13	120.5 (9)
C7—C2—C3	122.9 (8)	H15—C15—C14	117.1 (5)
H3—C3—C2	120.9 (5)	C16—C15—C14	125.7 (8)
C4—C3—C2	118.2 (8)	C16—C15—H15	117.1 (5)
C4—C3—H3	120.9 (6)	C15—C16—O4	124.8 (8)
H4—C4—C3	120.5 (6)	C17—C16—O4	115.9 (8)
C5—C4—C3	119.1 (9)	C17—C16—C15	119.2 (8)
C5—C4—H4	120.5 (6)	H17a—C17—C16	109.5
H5—C5—C4	118.2 (6)	H17b—C17—C16	109.5
C6—C5—C4	123.7 (9)	H17b—C17—H17a	109.5
C6—C5—H5	118.2 (5)	H17c—C17—C16	109.5
H6—C6—C5	121.1 (5)	H17c—C17—H17a	109.5
C7—C6—C5	117.8 (8)	H17c—C17—H17b	109.5
			
Co1—O1—C9—C8	170.8 (7)	N1—C1—S1—C2	0.6 (7)
Co1—O1—C9—C10	−11.4 (8)	N1—C7—C2—C3	−179.8 (8)
Co1—O2—C11—C10	2.4 (7)	N1—C7—C6—C5	−179.4 (9)
Co1—O2—C11—C12	−178.2 (7)	N2—C1—S1—C2	−179.5 (8)
Co1—O3—C14—C13	165.0 (8)	N2—C1—N1—C7	178.9 (9)
Co1—O3—C14—C15	−14.8 (8)	C1—S1—C2—C3	179.0 (7)
Co1—O4—C16—C15	22.8 (7)	C1—S1—C2—C7	0.3 (5)
Co1—O4—C16—C17	−161.0 (8)	C1—N1—C7—C2	1.4 (8)
Co1—N1—C1—S1	173.4 (5)	C1—N1—C7—C6	−179.0 (7)
Co1—N1—C1—N2	−6.5 (7)	C2—C3—C4—C5	1.1 (12)
Co1—N1—C7—C2	−173.2 (7)	C2—C7—C6—C5	0.1 (9)
Co1—N1—C7—C6	6.4 (8)	C3—C2—C7—C6	0.6 (12)
S1—C1—N1—C7	−1.2 (7)	C3—C4—C5—C6	−0.4 (12)
S1—C2—C3—C4	−179.7 (10)	C4—C3—C2—C7	−1.2 (12)
S1—C2—C7—N1	−1.0 (7)	C4—C5—C6—C7	−0.2 (11)
S1—C2—C7—C6	179.4 (6)	C8—C9—C10—C11	−178.3 (9)
O1—C9—C10—C11	3.8 (11)	C9—C10—C11—C12	−178.3 (10)
O2—C11—C10—C9	1.1 (11)	C13—C14—C15—C16	174.0 (9)
O3—C14—C15—C16	−6.2 (11)	C14—C15—C16—C17	−174.9 (10)
O4—C16—C15—C14	1.3 (12)		

Symmetry code: (i) −*x*+2, −*y*+1, −*z*+2.

### (2-Aminobenzothiazole-κN3)aquabis(4-oxopent-2-en-2-olato-κ2O,O')cobalt(II) Hydrogen-bond geometry (Å, º)

*Cg*4 and *Cg*5 are the centroids of the C2–C7 ring and the N1/S1/C1–C7 ring system, respectively.

**Table:**

*D*—H···*A*	*D*—H	H···*A*	*D*···*A*	*D*—H···*A*
O1*W*—H1*Wa*···O2^ii^	0.92 (2)	1.98 (3)	2.808 (8)	148 (5)
O1*W*—H1*Wb*···O4^ii^	0.92 (1)	1.97 (3)	2.820 (8)	154 (5)
N2—H2*b*···O1^iii^	0.86 (1)	2.29 (1)	3.074 (10)	151 (1)
C6—H6···S1^iv^	0.93 (1)	2.85 (1)	3.568 (10)	135 (1)
N2—H2*a*···O2	0.86 (1)	2.48 (1)	3.060 (10)	126 (1)
N2—H2*a*···O4	0.86 (1)	2.38 (1)	3.028 (10)	132 (1)
C6—H6···O3	0.93 (1)	2.53 (1)	3.189 (11)	128 (1)

Symmetry codes: (ii) −*x*+1, −*y*, −*z*+1; (iii) *x*+1, *y*, *z*; (iv) *x*−1, *y*, *z*.
